# Behavioral and functional aspects among public and private middle school students

**DOI:** 10.1590/2317-1782/e20250009en

**Published:** 2026-06-19

**Authors:** Maria Júlia Amaral Abranches de Almeida, Graziela Nunes Alfenas Fernandes, Stela Maris Aguiar Lemos

**Affiliations:** 1 Departamento de Fonoaudiologia, Faculdade de Medicina, Universidade Federal de Minas Gerais – UFMG - Belo Horizonte (MG), Brasil.

**Keywords:** Adolescent, Behavior, International Classification of Functioning, Disability and Health, Quality of Life, Mental Health

## Abstract

**Purpose:**

To investigate the relationship between behavioral aspects (difficulties and abilities) and the functioning of middle school students, considering variables such as grade in school, sex, age, economic class, and type of school.

**Methods:**

Observational, cross-sectional study with 157 students aged 11 to 14 years, enrolled in public and private schools. The instruments used were participant characterization, Brazilian Economic Classification Criteria (CCEB), Strengths and Difficulties Questionnaire (SDQ-Por), and the International Classification of Functioning, Disability, and Health (ICF), applied via Google Forms. The analysis was performed using SPSS software (version 25.0).

**Results:**

Most participants had a normal classification on the SDQ scales and were evaluated as "no difficulties" in the ICF categories. There were statistically significant associations between SDQ emotional symptoms and ICF codes d240, d710, and d750, as well as between conduct problems and ICF codes d240, d710, d720, and d750. Symptoms of hyperactivity were associated with sex and ICF codes d240, d710, and d720, while peer relationship problems were associated with codes d750 and d710. Prosocial behavior was associated with sex and ICF codes d240, d710, and d750. The total SDQ score was associated with the ICF codes.

**Conclusion:**

The study highlights the importance of understanding the behavioral and functional specificities of adolescents to promote a better socio-emotional quality of life among them.

## INTRODUCTION

Adolescents’ self-awareness can be determined by the interactions between internal and external factors, including the family context and the educational environment^(1)^. Concerning health, the psychological, neurological, and psychiatric conditions are among the main causes of disability in adolescents. According to 2021 research by the United Nations Children's Fund (UNICEF), Brazilian adolescents presented the second-worst index of optimism regarding the future. The study revealed that 48% of adolescents frequently feel nervous, worried, or anxious, while 22% report frequently feeling depressed or unmotivated to perform daily activities.

On the other hand, Brazil occupies the second position among the countries in which the young people interviewed most believe in the transformative power of education to promote social change^(2)^. According to the Pan American Health Organization (PAHO), half of mental health conditions begin at age 14, although most are neither diagnosed nor treated^(3)^. For this reason, neglecting mental health in this age group can lead to negative consequences in adulthood, causing damage to physical and mental health and limiting future opportunities^(3,4)^.

Observing adolescent behavior allows for the identification of possible behavioral and psychological conditions, favoring early intervention^(1)^. Behavioral aspects in adolescent students are mostly noticed by parents or guardians and by teachers at school, being directly related to learning difficulties^(5)^. Furthermore, compromised mental health negatively impacts not only the individual but also the school, family, and social contexts^(6)^.

Regarding functioning, the World Health Organization (WHO) defined the International Classification of Functioning, Disability, and Health (ICF) as an instrument to classify the health status of individuals, considering functional and disabling aspects^(7)^. The ICF adopts the biopsychosocial model, based on a multidirectional relationship between the biological, psychological, and social spheres, allowing interaction between these levels^(8)^.

Although research widely uses the ICF, its application in clinical practice is still limited. Healthcare professionals mostly adopt the biomedical model, centered on the patient's physical conditions, disregarding psychosocial aspects^(9)^. The ICF is a multidimensional tool applied in health, education, and research, promoting an integrated view of functioning and disability. Its use is essential for comprehensive assessments, enabling effective interventions that minimize limitations and promote the well-being of children and adolescents^(10)^. Applying this classification in this population allows a comprehensive understanding of health and supports individual and social strategies aimed at reducing negative impacts among them^(9)^.

Although the literature presents different age ranges for defining adolescence, the WHO has defined it in developing countries as the period between 10 and 19 years of age, subdividing it into "young adolescents" from 10 to 14 years of age and "older adolescents" from 15 to 19 years of age^(11)^. The Brazilian Statute of Children and Adolescents (ECA) establishes the age range of 12 to 18 years as a normative reference^(12)^. In this sense, the inclusion of individuals from the age of 11 as part of the case study is justified because it is a stage that, according to international parameters, is already considered the beginning of adolescence. This decision is relevant because it captures initial manifestations of behavioral and functional aspects that may emerge in this early period and that are often determinant for mental health and overall development. Furthermore, when considering young adolescents, it increases the possibility of early identification of psychological, neurological, and social conditions that may negatively impact their functioning and well-being.

Understanding whether the behavioral aspects of middle school students are influenced by socioeconomic variables (e.g., economic class, sex, and age) and school factors is fundamental to broadening the understanding of child and adolescent development. It is also relevant to map their functional aspects, using a mental health assessment instrument based on the ICF to provide more assertive and targeted interventions. Therefore, this study aimed to verify the association of behavioral aspects (difficulties and abilities) and functioning with the grade in school, sex, age, economic class, and type of school (public or private) in middle school students.

## METHODS

This is an observational, analytical, cross-sectional study with 157 adolescent students enrolled in public and private middle schools located in the Central-South and Northeast regions of the municipality of Belo Horizonte, Minas Gerais, Brazil.

This study was approved by the Research Ethics Committee of the Federal University of Minas Gerais (COEP/UFMG), under approval number 4,446,496.

The sample was non-probabilistic, composed of adolescents aged 11 to 14 years, enrolled in public and private middle schools. The institutions were selected by convenience.

The inclusion criteria were adolescents whose parents or guardians agreed to the informed consent form, who signed an informed assent form, and who fully responded to the proposed assessment instruments. Adolescents with evidence of cognitive, neurological, or psychiatric alterations that prevented participation in the research were excluded, based on information provided by the school team during the participant selection process. The identification was based on diagnoses already documented by the institution, previously reported by the parents/guardians to the school.

Participants were recruited through an invitation letter, sent by the school and the researchers. Parents or guardians signed an informed consent form, and students signed an informed assent form. Data were collected between August 2021 and September 2022, in a hybrid format, using internationally validated research instruments transcribed into forms created on Google Forms.

The following instruments were used: participant characterization questionnaire, Brazilian Economic Classification Criteria (CCEB) of the Brazilian Association of Market Research (2022), Strengths and Difficulties Questionnaire (SDQ-Por)^(13)^, and the ICF^(7)^. The characterization questionnaire aimed to obtain information such as age, sex, grade in school, and type of school they attended.

The CCEB analyzed socioeconomic aspects through the householder’s purchasing power and education level, distributed in classes A1, A2, B1, B2, C, D, and E, with A1 being the highest purchasing power and E the lowest.

The SDQ-Por, translated and validated for Brazil^(14)^, assessed the psychopathological capabilities and difficulties of children and adolescents aged 4 to 16 years. The instrument, developed for screening child and adolescent mental health, is composed of five scales: prosocial behavior, hyperactivity, emotional symptoms, conduct problems, and peer problems. Each scale has a score interpreted according to the established cutoff. The final score was obtained by summing the scales.

The ICF, developed by the WHO, is based on a biopsychosocial model that encompasses health components at the physical, psychological, and social levels. Its structure is three-dimensional and includes biomedical, psychological, and social aspects.

This study described the participants' functioning based on the "Activities and Participation" categories, using performance qualifiers, and the "Environmental Factors" categories, with facilitator and barrier qualifiers.

The ICF categories were not used in data collection, having been previously selected based on their relationship with the domains assessed by the SDQ. After selection, the SDQ results were coded according to the corresponding qualifiers. Only performance qualifiers were used, since the SDQ is an instrument answered by caregivers based on observation of the adolescent's behavior in their usual environment. The qualifiers used were: .0 (no difficulty) and .8 (unspecified difficulty), without determining the degree of difficulty. Four categories were selected: handling stress and other psychological demands (d240), complex interpersonal interactions (d720), informal social relationships (d750), and basic interpersonal interactions (d710). The d240 code was analyzed in two aspects: emotional symptoms and hyperactivity.

In the first stage of the analysis, the response variable was composed of the participants' abilities and difficulties (behavioral aspects). The explanatory variables were economic classification, grade in school, type of school, sex, and age. In the second stage, the response variables were the four ICF "Activities and Participation" categories, coded from the SDQ results, maintaining the conceptual equivalence of the qualifiers used, ensuring correspondence between the ICF categories and the behavioral aspects evaluated.

A descriptive analysis of the data was performed to meet the study objective, using the frequency distribution of categorical variables and analysis of measures of central tendency and dispersion of continuous variables. Fisher's exact test and Pearson's chi-square test were used for association analyses. SPSS software, version 25.0, was used for data entry, processing, and analysis.

## RESULTS

The total sample consisted of 157 individuals, with a mean age of 12.62 years, a standard deviation of 1.08, and a median of 13.00 years. The majority were female (55.4%). Most were in the 7^th^ grade (28.7%), followed by the 6^th^ grade (26.1%) and the 8^th^ grade (25.5%); the 9^th^ grade had the lowest representation (19.7%). Most adolescents were enrolled in private schools (79.0%).

Also, most adolescents belonged to CCEB class A1 (Brazilian Association of Market Research, 2022) (64.5%), followed by classes A2 (15.8%), B2 (8.5%), B1 (7.8%), and C1 (2.1%); classes C2 (0.7%) and D (0.7%) were the least represented.

The descriptive analysis of the SDQ showed that most participants’ responses in the questionnaires designated a normal classification on all scales: “Emotional Symptoms” (80.8%), “Conduct Problems” (79.6%), “Hyperactivity” (72.0%), “Peer Problems” (80.3%), and “Prosocial Behavior” (89.8%). In the total SDQ score, which indicates the participants' mental health difficulties, 72.0% were classified as normal, indicating good mental health. Furthermore, 14.6% of the participants presented a borderline result, and 13.4% obtained a score classified as abnormal.

The descriptive analysis of the ICF (performance) codes demonstrated that, in all of them, most participants were classified as "no difficulty" – i.e., they classified themselves as capable of performing the domains effectively, independently, and functionally, being: d240 – emotional symptoms (80.9%), d720 – complex interpersonal interactions (79.6%), d240 – hyperactivity (71.3%), d750 – informal social relationships (80.3%), and d710 – basic interpersonal interactions (89.8%).

The SDQ classification was grouped as a) abnormal and b) borderline/normal, for better association analysis. The CCEB groups were grouped as follows: a) A1/A2 = A; b) B1/B2 = B; and c) C1/C2/D = C-D.

Table 1 presents the association analysis between the SDQ scales Emotional Symptoms and Conduct Problems with sociodemographic data and ICF codes (performance), using Fisher's exact and Pearson's chi-square tests. "Emotional Symptoms" were statistically significantly associated with the ICF codes d240 emotional symptoms (p = 0.001), d720 complex interpersonal interactions (p = 0.001), d240 hyperactivity (p = 0.001), and d750 informal social relationships (p = 0.001). Also, “Conduct Problems” were statistically significantly associated with codes d240 emotional symptoms (p = 0.001), d720 (p = 0.001), d750 informal social relationships (p = 0.011), and d710 basic interpersonal interactions (p = 0.001). The other analyses had no statistically significant results.

**Table 1:** Analysis of the association between the SDQ emotional symptoms and conduct problems scales and sociodemographic data and ICF

**Variables**	**Emotional Symptoms**			**Conduct Problems**		
	**Abnormal N (%)**	**Borderline/Normal N (%)**	**p-value**	**Abnormal N (%)**	**Borderline/Normal N (%)**	**p-value**
**Sex**						
Females	11 (73.3)	76 (53.5)	0.142^1^	10 (62.5)	77 (54.6)	0.547^2^
Males	4 (26.7)	66 (46.5)		6 (37.5)	64 (45.4)	
Total	15 (100.0)	142 (100.0)		16 (100.0)	141 (100.0)	
**Grade in school**						
6^th^ grade	2 (13.3)	39 (27.5)	0.233^2^	3 (18.7)	38 (27.0)	0.550^2^
7^th^ grade	7 (46.7)	38 (26.8)		7 (43.8)	38 (27.0)	
8^th^ grade	2 (13.3)	38 (26.8)		3 (18.8)	37 (26.2)	
9^th^ grade	4 (26.7)	27 (19.0)		3 (18.7)	28 (19.9)	
Total	15 (100.0)	142 (100.0)		16 (100.0)	141 (100.0)	
**Type of school**						
Public	2 (13.3)	31 (21.8)	0.442^1^	6 (37.5)	27 (19.1)	0.088^1^
Private	13 (86.7)	111 (78.2)		10 (62.5)	114 (80.9)	
Total	15 (100.0)	142 (100.0)		16 (100.0)	141 (100.0)	
**CCEB**						
A	13 (86.7)	100 (79.4)	0.679^2^	10 (66.7)	103 (81.7)	0.138^2^
B	2 (13.3)	21 (16.7)		5 (33.3)	18 (14.3)	
C-D	0 (0.0)	5 (4.0)		0 (0.0)	5 (4.0)	
Total	15 (100.0)	126 (100.0)		16 (100.0)	126 (100.0)	
**d240 - ES**						
No difficulty	0 (0.0)	127 (89.4)	0.001^1^*	8 (50.0)	119 (84.4)	0.001^2^*
With difficulties	15 (100.0)	15 (10.6)		8 (50.0)	22 (15.6)	
Total	15 (100.0)	142 (100.0)		16 (100.0)	141 (100.0)	
**d720**						
No difficulty	7 (46.7)	118 (83.1)	0.003^*^	0 (0.0)	125 (88.7)	0.001^1^*
With difficulties	8 (53.3)	24 (16.9)		16 (100.0)	16 (11.3)	
Total	15 (100.0)	142 (100.0)		16 (100.0)	141 (100.0)	
**d240 - Hyper**						
No difficulty	9 (60.0)	103 (72.5)	0.307^2^	7 (43.8)	105 (74.5)	0.017^2^*
With difficulties	6 (40.0)	39 (27.5)		9 (56.2)	36 (25.5)	
Total	15 (100.0)	142 (100.0)		16 (100.0)	141 (100.0)	
**d750**						
No difficulty	5 (33.3)	108 (76.1)	0.001^2^*	9 (56.3)	117 (83.0)	0.011^2^*
With difficulties	10 (66.7)	34 (23.9)		7 (43.7)	24 (17.0)	
Total	15 (100.0)	142 (100.0)		16 (100.0)	141 (100.0)	
**d710**						
No difficulty	11 (73.3)	130 (91.5)	0.051^1^	10 (62.5)	131 (92.9)	0.001^2^*
With difficulties	4 (26.7)	12 (8.5)		6 (37.5)	10 (7.1)	
Total	15 (100.0)	142 (100.0)		16 (100.0)	141 (100.0)	

1 Fisher’s exact test;

2 Pearson chi-square test

* p-value ≤ 0.05

**Caption:** N = number of individuals, varying due to missing data; CCEB = Brazilian Economic Classification Criteria; ES = Emotional Symptoms; Hyper = Hyperactivity; d240 = Handling stress and other psychological demands; d720 = Complex interpersonal interactions; d750 = Informal social relationships; d710 = Basic interpersonal interactions

Table 2 presents the association between the SDQ “Hyperactivity” and “Peer Problems”, sociodemographic data, and ICF codes, analyzed using Pearson's chi-square and Fisher's exact tests. The results indicated a statistically significant association between hyperactivity and sex (p = 0.024), with a higher percentage of borderline/normal classification among female students. Significant associations were also observed between hyperactivity and the ICF codes: d240 – emotional symptoms (p = 0.001), d720 – complex interpersonal interactions (p = 0.001), d240 – hyperactivity (p = 0.001), and d710 – basic interpersonal interactions (p = 0.013). “Peer Problems” were significantly associated with d240 – emotional symptoms (p = 0.048) and d750 – informal interpersonal relationships (p = 0.001). The other associations had no statistical significance.

**Table 2:** Analysis of the association between the SDQ hyperactivity and peer problems scales and sociodemographic data and ICF codes

**Variables**	**Hyperactivity**			**Peer Problems**		
	**Abnormal N (%)**	**Borderline/Normal N (%)**	**p-value**	**Abnormal N (%)**	**Borderline/Normal N (%)**	**p-value**
**Sex**						
Females	19 (76.0)	68 (51.5)	0.024^1^*	3 (60.0)	84 (55.3)	0.834^2^
Males	6 (24.0)	64 (48.5)		2 (40.0)	68 (44.7)	
Total	25 (100.0)	132 (100.0)		5 (100.0)	152 (100.0)	
**Grade in school**						
6^th^ grade	6 (24.0)	35 (26.5)	0.988^1^	0 (0.0)	41 (27.00)	0.330^1^
7^th^ grade	7 (28.0)	38 (28.8)		3 (60.0)	42 (27.6)	
8^th^ grade	7 (28.0)	33 (25.0)		0 (0.0)	40 (26.3)	
9^th^ grade	5 (20.0)	26 (19.7)		2 (40.0)	29 (19.1)	
Total	25 (100.0)	132 (100.0)		5 (100.0)	152 (100.0)	
**Type of school**						
Public	8 (32.0)	25 (18.9)	0.142^1^	2 (40.0)	31 (20.4)	0.290^2^
Private	17 (68.0)	108 (81.1)		3 (60.0)	121 (79.6)	
Total	25 (100.0)	132 (100.0)		5 (100.0)	152 (100.0)	
**CCEB**						
A	15 (68.2)	98 (82.4)	0.073^1^	2 (50.0)	111 (81.0)	0.051^1^
B	7 (31.8)	16 (13.4)		1 (25.0)	22 (16.1)	
C-D	0 (0.0)	5 (4.2)		1 (25.0)	4 (2.9)	
Total	25 (100.0)	119 (100.0)		4 (100.0)	137 (100.0)	
**d240 - ES**						
No difficulty	13 (52.0)	114 (86.4)	0.001^1 *^	2 (40.0)	125 (82.2)	0.048^2^*
With difficulties	12 (48.0)	18 (13.6)		3 (60.0)	27 (17.8)	
Total	25 (100.0)	132 (100.0)		5 (100.0)	152 (100.0)	
**d720**						
No difficulty	13 (52.0)	112 (84.8)	0.001^1^*	3 (60.0)	122 (80.3)	0.269^2^
With difficulties^1^	12 (48.0)	20 (15.2)		2 (40.0)	30 (19.7)	
Total	25 (100.0)	132 (100.0)		5 (100.0)	152 (100.0)	
**d240 - Hyper**						
No difficulty	0 (0.0)	112 (84.8)	0.001^2^*	3 (60.0)	109 (71.7)	0.625^2^
With difficulties	25 (100.0)	20 (15.2)		2 (40.0)	43 (28.3)	
Total	25 (100.0)	132 (100.0)		5 (100.0)	152 (100.0)	
**d750**						
No difficulty	17 (68.0)	109 (82.6)	0.093^1^	0 (0.0)	126 (82.9)	0.001^2^*
With difficulties	8 (32.0)	23 (17.4)		5 (100.0)	26 (17.1)	
Total	25 (100.0)	132 (100.0)		5 (100.0)	152 (100.0)	
**d710**						
No difficulty	19 (76.0)	122 (92.4)	0.013*	3 (60.0)	138 (90.8)	0.081^2^
With difficulties	6 (24.0)	10 (7.6)		2 (40.0)	14 (9.2)	
Total	25 (100.0)	132 (100.0)		5 (100.0)	152 (100.0)	

1 Pearson chi-square test;

2 Fisher’s exact test

* p-value ≤ 0.05

**Caption:** N = number of individuals, varying due to missing data; CCEB = Brazilian Economic Classification Criteria; ES = Emotional Symptoms; Hyper = Hyperactivity; d240 = Handling stress and other psychological demands; d720 = Complex interpersonal interactions; d750 = Informal social relationships; d710 = Basic interpersonal interactions

Finally, in Table 3, an association analysis was performed between the “Prosocial Behavior” and SDQ total scales, using Fisher's exact and Pearson's chi-square tests, with sociodemographic data and ICF codes. A statistically significant association was found between “Prosocial Behavior” and sex (p = 0.023), with a higher percentage of borderline/normal results among female participants, and with the ICF codes d240 – hyperactivity (p = 0.007), d750 - informal social relationships (p = 0.049), and d710 - basic interpersonal interactions (p = 0.001).

**Table 3:** Analysis of the association between the Prosocial Behavior scale and Total SDQ and sociodemographic data and ICF codes

**Variables**	**Prosocial behavior**			**Total SDQ**		
	**Abnormal N (%)**	**Borderline/Normal N (%)**	**p-value**	**Abnormal N (%)**	**Borderline/Normal N (%)**	**p-value**
**Sex**						
Females	1 (12.5)	86 (57.7)	0.023^1^*	15 (71.4)	72 (52.9)	0.157^1^
Males	7 (87.5)	63 (42.3)		6 (28.6)	64 (47.1)	
Total	8 (100.0)	149 (100.0)		21 (100.0)	136 (100.0)	
**Grade in school**						
6^th^ grade	2 (25.0)	39 (26.2)	0.236^2^	3 (14.3)	38 (27.9)	0.334^2^
7^th^ grade	0 (0.0)	45 (30.2)		8 (38.1)	37 (27.2)	
8^th^ grade	3 (37.5)	37 (24.8)		4 (19.0)	36 (26.5)	
9^th^ grade	3 (37.5)	28 (18.8)		6 (28.6)	25 (18.4)	
Total	8 (100.0)	149 (100.0)		21 (100.0)	136 (100.)	
**Type of school**						
Public	3 (37.5)	30 (20.1)	0.366^1^	5 (23.8)	28 (20.6)	0.736^2^
Private	5 (62.5)	119 (79.9)		16 (76.2)	108 (79.4)	
Total	8 (100.0)	149 (100.0)		21 (100.0)	136 (100.0)	
**CCEB**						
A	4 (50.0)	109 (82.0)	0.074^2^	14 (77.8)	99 (80.5)	0.552^2^
B	3 (37.5)	20 (15.0)		4 (22.2)	19 (15.4)	
C-D	1 (12.5)	4 (3.0)		0 (0.0)	5 (4.1)	
Total	8 (100.0)	133 (100.0)		21 (100.0)	123 (100.0)	
**d240 – ES**						
No difficulty	5 (62.5)	122 (81.9)	0.179^1^	5 (23.8)	122 (89.7)	0.001^2^*
With difficulties	3 (37.5)	27 (18.1)		16 (76.2)	14 (10.3)	
Total	8 (100.0)	149 (100.0)		21 (100.0)	136 (100.0)	
**d720**						
No difficulty	5 (62.5)	120 (80.5)	0.207^1^	7 (33.3)	118 (86.8)	0.001^2^*
With difficulties	3 (37.5)	29 (19.5)		14 (66.7)	18 (13.2)	
Total	8 (100.0)	149 (100.0)		21 (100.0)	136 (100.0)	
**d240 - Hyper**						
No difficulty	2 (25.0)	110 (73.8)	0.007^1 *^	7 (33.3)	105 (77.2)	0.001^2^*
With difficulties	6 (75.0)	39 (26.2)		14 (66.7)	31 (22.8)	
Total	8 (100.0)	149 (100.0)		21 (100.0)	136 (100.0)	
**d750**						
No difficulty	4 (50.0)	122 (81.9)	0.049^1^*	7 (33.3)	119 (87.5)	0.001^2^*
With difficulties	4 (50.0)	27 (18.1)		14 (66.7)	17 (12.5)	
Total	8 (100.0)	149 (100.0)		21 (100.0)	136 (100.0)	
**d710**						
No difficulty	0 (0.0)	141 (94.6)	0.001^1^*	13 (61.9)	128 (94.1)	0.001^2^*
With difficulties	8 (100.0)	8 (5.4)		8 (38.1)	8 (5.9)	
Total	8 (100.0)	149 (100.0)		21 (100.0)	136 (100.0)	

1 Fisher’s exact test;

2 Pearson chi-square test

* p-value ≤ 0.05

**Caption:** N = number of individuals, varying due to missing data; CCEB = Brazilian Economic Classification Criteria; ES = Emotional Symptoms; Hyper = Hyperactivity; d240 = Handling stress and other psychological demands; d720 = Complex interpersonal interactions; d750 = Informal social relationships; d710 = Basic interpersonal interactions

The total SDQ score was statistically significantly associated with the ICF codes d240 – emotional symptoms (p = 0.001), d720 - complex interpersonal interactions (p = 0.001), d240 – hyperactivity (p = 0.001), d720 (p = 0.001), d240 – hyperactivity (p = 0.001), d750 - informal social relationships (p = 0.001), and d710 - basic interpersonal relationships (p = 0.001).

## DISCUSSION

According to the literature, the clinical application of the SDQ has been carried out in several countries. However, normative significance data are limited to a few populations and age groups^(15)^. A reliable and valid assessment of the behavioral aspects of young people is crucial for the early detection and identification of clinical cases in the childhood and adolescence phase^(16)^. However, the questionnaire comprises only five scales, not covering all the highly complex psychosocial aspects, especially in adolescence, when they are not yet fully aware of their actions^(17,18)^.

The SDQ Emotional Symptoms scale was associated with d240 - emotional symptoms and d240 - hyperactivity, d720, and d750. According to the literature, adolescents with high indicators of emotional distress concomitantly present significant difficulties in building and maintaining social relationships, as well as in emotional and behavioral self-regulation^(19,20)^.

The association between Conduct Problems and the codes d240 - emotional symptoms, d710, d720, and d750 demonstrates that students with higher scores in conduct problems show a correlation with significant difficulties in interpersonal interactions, both in the basic sphere and in more complex social relationships. Externalizing behaviors, such as aggressiveness, disobedience, and impulsivity, are directly associated with deficits in socio-emotional skills, harming informal relationships and the ability to build and maintain healthy interpersonal interactions^(21)^. These difficulties are not limited to social interaction; they also significantly impact the adolescent's ability to regulate their emotions and interact functionally in basic interpersonal contexts^(22)^.

It was observed that more girls than boys present symptoms of hyperactivity. According to the literature, female children and adolescents express symptoms of hyperactivity and impulsivity through speech and actions, unlike boys, who are more rebellious and oppositional^(23)^. Since girls have a higher prevalence of anxious and depressive traits, they may experience greater emotional fluctuations, especially in the age range studied^(23)^. Moreover, recent studies indicate that the prevalence of Attention-Deficit/Hyperactivity Disorder (ADHD) is similar between men and women^(24)^. Also, this scale was correlated with the categories d240 - emotional symptoms, d240 - hyperactivity, d710, and d720. Recent studies indicate that adolescents with high scores on the hyperactivity scale have greater difficulties in managing emotional and cognitive demands^(19,20)^. Impulsivity and restlessness hinder more sophisticated social skills, such as conflict resolution, empathy, negotiation, and maintaining lasting affective bonds^(21)^. Adolescents may also have difficulties even in the simplest everyday interactions, such as initiating or maintaining a conversation and respecting turn-taking, significantly compromising their social integration and performance in the school and family environment^(22)^.

The Peer Problems scale was statistically significantly associated with d240 - emotional symptoms and d750. Current studies show that adolescents with high scores in relationship problems tend to present associated emotional difficulties, such as social anxiety, insecurity, and low frustration tolerance, aspects directly related to the inability to manage stress and daily emotional demands^(20)^. They also show impairments in essential skills for social interaction, expressing themselves appropriately, or understanding facial expressions and basic social cues, revealing a dysfunctional social functioning pattern^(21)^.

The study in question revealed worse results for males on the Prosocial Behavior scale. These aspects are also influenced by sociocultural factors and socialization practices. In the Brazilian context, parental practices tend to more frequently reinforce behaviors of care, empathy, and collaboration in girls. On the other hand, boys are more frequently directed to suppress emotions and adopt resistant postures^(25)^. The socialization process from childhood, guided by cultural norms and gender stereotypes, shapes how girls and boys deal with emotions and relationships, directly influencing the development of socio-emotional skills and prosocial behaviors^(26)^. There was also statistical significance in the behavior scale and the codes d40 - hyperactivity, d750 - informal social relationships, and d710 - basic interpersonal interactions. Adolescents with low scores in prosocial behavior have difficulties interacting and face challenges in emotional management, such as anxiety, frustration, and impulsivity. This compromises their social inclusion and well-being, highlighting that emotional balance and functional competence in relationships are fundamental for socio-emotional development^(20-22)^.

The analysis of the total SDQ score showed a significant correlation with all the ICF codes analyzed. The literature highlights that adolescents with high SDQ scores tend to have greater difficulties in dealing with stress and emotional demands, which negatively impacts their social interactions^(25)^. This condition generates impairments in emotional management, self-regulation, and social interactions, manifesting as low tolerance to frustration, constant stress, difficulty in impulse control, and difficulty in developing interactive social skills, empathy, conflict resolution, and maintaining bonds^(19,20)^.

According to the literature, the ICF has effective applicability in studies with samples of children and adolescents. This is because the ICF guarantees a standardized and biopsychosocial model that encompasses the social, biological, and psychological spheres. This model allows for a broader identification of disabilities that may arise throughout development^(27,28)^.

The literature shows that, overall, adolescents with language disorders have SDQ scores classified as abnormal^(29)^. Thus, the association of SDQ scales with ICF categories demonstrated that adolescents with a normal classification on the questionnaire did not present performance difficulties. In this sense, a Canadian longitudinal study with adolescents and young people with chronic conditions explored the ICF, its functional components and contextual factors, providing evidence of the relationship between emotional and functional aspects and quality of life^(30)^.

The Pan American Health Organization (PAHO) highlights that school interventions are essential preventive strategies to combat adverse mental health conditions. Among the recommended actions are the implementation of organizational changes that promote a safe and positive psychological environment, the inclusion of teachings on mental health, and the development of life skills, the training of professionals to identify and manage basic suicide risks, and the creation of prevention programs specifically aimed at adolescents vulnerable to mental health problems^(31)^.

This study has some limitations, the first being the sample design and configuration, without an equitable distribution of adolescents from public and private schools. Thus, the predominance of participants from private schools over public schools may have influenced the results of this analysis. Consequently, the distribution of participants according to economic class may also have been affected, so that convenience sampling prevents the generalization of the findings to other contexts.

As advances, the study integrated the SDQ with the ICF, ensuring multidimensional assessment and providing a more comprehensive view of behavioral aspects and functioning in the school and social environment. Moreover, the identification of categories associated with the ICF (prosocial behavior, hyperactivity, emotional, conduct, and peer problems) facilitates the formulation of more targeted interventions. This study also enables the use of the ICF at school and creates a starting point for further research involving this age group, allowing the exploration of different contexts, populations, and interventions based on this approach.

## CONCLUSION

Given the results, the integration between the SDQ and the ICF proves to be an effective strategy for comprehensively understanding the relationships between the emotional, behavioral, and functional aspects of adolescents at school. The associations found reinforce that emotional and behavioral difficulties directly impact social functioning, stress management, and the quality of interpersonal interactions.

These findings demonstrate similarities with research conducted in Brazil and other countries, especially regarding the use of the ICF, to understand behavioral and functional aspects in adolescents. Thus, it is evident that understanding these aspects and their particularities is essential to ensure better emotional and social quality of life for this age group.
