# Supplementary material for: Behavioral and functional aspects among public and private middle school students
Source: Codas. 2026 Jun 19;38(4):e20250009. doi: 10.1590/2317-1782/e20250009en (PMC13322491; doi:10.1590/2317-1782/e20250009en)
Supplement: Translation

# Translation: Aspectos comportamentais e funcionais em escolares do Ensino Fundamental de escolas públicas e privadas

**DOI:** 10.1590/2317-1782/e20250009pt

**Authors:** Maria Júlia Amaral Abranches de Almeida, Graziela Nunes Alfenas Fernandes, Stela Maris Aguiar Lemos

**Affiliations:** 1 Departamento de Fonoaudiologia, Faculdade de Medicina, Universidade Federal de Minas Gerais – UFMG - Belo Horizonte (MG), Brasil.

**Keywords:** Adolescente, Comportamento, Classificação Internacional de Funcionalidade, Incapacidade e Saúde, Qualidade de Vida, Saúde Mental

## Abstract

**Objetivo:**

Investigar a relação entre aspectos comportamentais (dificuldades e capacidades) e a funcionalidade de estudantes do Ensino Fundamental II, considerando variáveis como ano escolar, sexo, idade, classe econômica e tipo de escola.

**Método:**

Estudo observacional e transversal com 157 escolares de 11 a 14 anos, matriculados em escolas públicas e privadas. Foram utilizados os instrumentos Caracterização dos Participantes, Critério de Classificação Econômica Brasil (CCEB), Strengths and Difficulties Questionnaire (SDQ-Por) e a Classificação Internacional de Funcionalidade, Incapacidade e Saúde (CIF), aplicados via Google Forms. A análise foi realizada com o software SPSS (versão 25.0).

**Resultados:**

A maioria dos participantes apresentou classificação normal nas escalas do SDQ e foi avaliada como “sem dificuldades” nas categorias da CIF. Houve associações estatisticamente significativas entre sintomas emocionais do SDQ e os códigos da CIF d240, d710 e d750, bem como entre problemas de conduta e os códigos CIF d240, d710, d720 e d750. Sintomas de hiperatividade apresentaram associação com sexo e os códigos CIF d240, d710 e d720, enquanto problemas de relacionamento com colegas se associaram aos códigos d750 e d710. Comportamento pró-social foi associado ao sexo e a códigos CIF d240, d710 e d750. O escore total do SDQ apresentou associação com os códigos CIF.

**Conclusão:**

Destaca-se a importância de compreender as especificidades comportamentais e funcionais dos adolescentes para promover uma melhor qualidade de vida socioemocional nessa faixa etária.

## INTRODUÇÃO

O autoconhecimento que um adolescente possui pode ser determinado pelas interações entre fatores internos e externos, incluindo o contexto familiar e o ambiente educacional^(1)^. No que diz respeito à saúde, condições psicológicas, neurológicas e psiquiátricas estão entre as principais causas de incapacidade em adolescentes. De acordo com uma pesquisa realizada pelo Fundo das Nações Unidas para a Infância (UNICEF) em 2021, os adolescentes brasileiros apresentaram o segundo pior índice de otimismo em relação ao futuro. O estudo revelou que 48% dos adolescentes frequentemente se sentem nervosos, preocupados ou ansiosos, enquanto 22% relatam sentir-se frequentemente deprimidos ou sem motivação para realizar atividades cotidianas.

Por outro lado, o Brasil ocupa a segunda posição entre os países nos quais os jovens entrevistados mais acreditam no poder transformador da educação para promover mudanças sociais^(2)^. Segundo a Organização Pan-Americana da Saúde (OPAS), metade das condições de saúde mental tem início aos 14 anos, embora a maioria não seja diagnosticada nem tratada^(3)^. Por esse motivo, negligenciar o estado de saúde mental nessa faixa etária pode acarretar consequências negativas na vida adulta, causando danos à saúde física e mental, além de limitar as oportunidades futuras^(3,4)^.

Observar o comportamento de adolescentes possibilita a identificação de possíveis condições comportamentais e psicológicas, favorecendo a intervenção precoce^(1)^. Os aspectos comportamentais em estudantes adolescentes são, em sua maioria, notados por pais ou responsáveis e por professores no ambiente escolar, estando diretamente relacionados às dificuldades de aprendizagem^(5)^. Ressalta-se que a saúde mental comprometida impacta negativamente não apenas o indivíduo, mas também os contextos escolar, familiar e social^(6)^.

No que se refere à funcionalidade, a Organização Mundial da Saúde (OMS) definiu a Classificação Internacional de Funcionalidade, Incapacidade e Saúde (CIF) como um instrumento para classificar a condição de saúde dos indivíduos, considerando aspectos funcionais e incapacitantes^(7)^. A CIF adota o modelo biopsicossocial, fundamentado em uma relação multidirecional entre as esferas biológica, psicológica e social, permitindo a interação entre esses níveis^(8)^.

Embora a CIF seja amplamente utilizada em pesquisas, sua aplicação na prática clínica ainda é limitada. Profissionais da área da saúde, em sua maioria, adotam o modelo biomédico, centrado nas condições físicas do paciente e desconsiderando aspectos psicossociais^(9)^. A CIF é uma ferramenta multidimensional aplicada nas áreas da saúde, educação e pesquisa, promovendo uma visão integrada da funcionalidade e incapacidade. Sua utilização é essencial para avaliações abrangentes, possibilitando intervenções eficazes que minimizem limitações e promovam o bem-estar de crianças e adolescentes^(10)^. A aplicação dessa classificação nessa população permite uma compreensão integral da saúde e subsidia estratégias individuais e sociais voltadas à redução dos impactos negativos nessa faixa etária^(9)^.

Embora a literatura apresente diferentes recortes etários para definição da adolescência, a Organização Mundial da Saúde (OMS), definiu, em regiões de países em desenvolvimento, a adolescência como o período entre os 10 e 19 anos, subdividindo-os em “jovens adolescentes” dos 10 aos 14 anos e “adolescentes mais velhos” dos 15 aos 19 anos^(11)^. Já o Estatuto da Criança e do Adolescente (ECA) estabelece a faixa etária dos 12 aos 18 anos como referência normativa^(12)^. Nesse sentido, a inclusão de indivíduos a partir dos 11 anos como parte da casuística justifica-se por se tratar de uma etapa que, segundo parâmetros internacionais, já é considerada início da adolescência. Tal decisão é relevante, pois permite captar manifestações iniciais de aspectos comportamentais e funcionais que podem emergir nesse período precoce e que, muitas vezes, são determinantes para a saúde mental e o desenvolvimento global. Além disso, ao contemplar adolescentes jovens, amplia-se a possibilidade de identificar precocemente condições psicológicas, neurológicas e sociais que podem impactar negativamente sua funcionalidade e bem-estar.

Compreender se os aspectos comportamentais de estudantes do Ensino Fundamental II sofrem influência de variáveis socioeconômicas, como classe econômica, sexo e idade, bem como de fatores escolares, é fundamental para ampliar o entendimento sobre o desenvolvimento infantojuvenil. Além disso, torna-se relevante mapear os aspectos funcionais dessa população, utilizando um instrumento de avaliação da saúde mental baseado na Classificação Internacional de Funcionalidade, Incapacidade e Saúde (CIF), a fim de proporcionar intervenções mais assertivas e direcionadas. Diante disso, o presente estudo teve como objetivo verificar a associação entre aspectos comportamentais (dificuldades e capacidades) e a funcionalidade, com as variáveis ano escolar, sexo, idade, classe econômica e tipo de escola (contexto público ou contexto privado) em escolares do Ensino Fundamental II.

## MÉTODO

Trata-se de um estudo observacional, analítico e transversal, realizado com 157 estudantes adolescentes matriculados no Ensino Fundamental II de escolas públicas e privadas localizadas nas regiões Centro-Sul e Nordeste do município de Belo Horizonte, Minas Gerais.

Este estudo foi aprovado pelo Comitê de Ética em Pesquisa da Universidade Federal de Minas Gerais (COEP/UFMG), sob o parecer nº 4.446.496.

A amostra foi não probabilística, composta por adolescentes com idades entre 11 e 14 anos, matriculados em escolas públicas e privadas de Ensino Fundamental II. As instituições foram selecionadas por conveniência.

Os critérios de inclusão foram: adolescentes cujos pais ou responsáveis concordaram com o Termo de Consentimento Livre e Esclarecido (TCLE), que assinaram o Termo de Assentimento Livre e Esclarecido (TALE) e que responderam integralmente aos instrumentos de avaliação propostos. Foram excluídos adolescentes que apresentassem evidências de alterações cognitivas, neurológicas ou psiquiátricas que impedissem a participação na pesquisa. Essa exclusão foi realizada a partir de informações fornecidas pela equipe escolar durante o processo de seleção dos participantes. A identificação baseou-se nos diagnósticos já documentados pela instituição, informados previamente pelos responsáveis à escola.

O recrutamento dos participantes foi realizado por meio de carta-convite, enviada pela escola e pelas pesquisadoras. Os pais ou responsáveis assinaram o TCLE, e os estudantes, o TALE. A coleta de dados ocorreu entre agosto de 2021 e setembro de 2022, de forma híbrida, por meio de instrumentos de pesquisa validados internacionalmente transcritos para formulários elaborados na plataforma Google Forms.

Foram utilizados os seguintes instrumentos: questionário de Caracterização dos Participantes; Critério de Classificação Econômica Brasil (CCEB) da Associação Brasileira de Empresas de Pesquisa (2022); *Strengths and Difficulties Questionnaire* (SDQ-Por)^(13)^; e a Classificação Internacional de Funcionalidade, Incapacidade e Saúde (CIF)^(7)^. O questionário de caracterização visou obter informações como idade, sexo, ano escolar e tipo de escola frequentada.

O Critério de Classificação Econômica Brasil (CCEB) analisou os aspectos socioeconômicos por meio do poder aquisitivo e do nível de escolaridade do chefe de família, distribuídos nas classes A1, A2, B1, B2, C, D e E, sendo A1 a de maior poder aquisitivo e E, a de menor.

O *Strengths and Difficulties Questionnaire* (SDQ-Por), traduzido e validado para o Brasil^(14)^, avaliou as capacidades e dificuldades psicopatológicas de crianças e adolescentes entre 4 e 16 anos. O instrumento, desenvolvido para triagem de saúde mental infanto-juvenil, é composto por cinco subescalas: comportamento pró-social, hiperatividade, problemas emocionais, problemas de conduta e problemas no relacionamento com pares. Cada subescala possui uma pontuação interpretada conforme os pontos de corte estabelecidos. A pontuação final foi obtida por meio da soma das subescalas.

A CIF, desenvolvida pela Organização Mundial da Saúde (OMS), fundamenta-se em um modelo biopsicossocial que contempla os componentes da saúde nos níveis físico, psicológico e social. Sua estrutura é tridimensional e inclui aspectos biomédicos, psicológicos e sociais.

Neste estudo, a funcionalidade dos participantes foi descrita com base nas categorias do componente “Atividades e Participação”, utilizando-se os qualificadores de desempenho, bem como nas categorias de “Fatores Ambientais”, com os qualificadores de facilitadores e barreiras.

As categorias da CIF não foram empregadas na coleta de dados, sendo previamente selecionadas com base em sua relação com os domínios avaliados pelo SDQ. Após a seleção, os resultados do SDQ foram codificados conforme os qualificadores correspondentes. Utilizaram-se apenas os qualificadores de desempenho, uma vez que o SDQ é um instrumento respondido pelos responsáveis com base na observação do comportamento do adolescente em seu ambiente habitual. Foram empregados os qualificadores: .0 (nenhuma dificuldade) e .8 (dificuldade não especificada), não sendo determinado o grau de dificuldade. Foram selecionadas quatro categorias: lidar com o estresse e outras exigências psicológicas (d240), interações interpessoais complexas (d720), relacionamentos interpessoais informais (d750) e interações interpessoais básicas (d710). O código d240 foi analisado em dois aspectos: sintomas emocionais e hiperatividade.

No primeiro momento da análise, a variável resposta foi composta pelas capacidades e dificuldades (aspectos comportamentais) dos participantes. As variáveis explicativas foram: classificação econômica, ano escolar, tipo de escola, sexo e idade. Na segunda etapa, as variáveis resposta foram as quatro categorias do componente “Atividades e Participação” da CIF, codificadas a partir dos resultados do SDQ mantendo a equivalência conceitual dos qualificadores utilizados, assegurando correspondência entre as categorias da CIF e os aspectos comportamentais avaliados.

Para atender ao objetivo do estudo foi realizada a análise descritiva dos dados, por meio da distribuição de frequência das variáveis categóricas e análise das medidas de tendência central e de dispersão das variáveis contínuas. Para as análises de associação foram utilizados os testes Exato de Fisher e Qui-quadrado de Pearson. Para entrada, processamento e análise dos dados foi utilizado o software SPSS, versão 25.0.

## RESULTADOS

A amostra total foi composta por 157 indivíduos, com média de idade de 12,62 anos, desvio padrão de 1,08 e mediana de 13,00 anos. A maioria era do sexo feminino (55,4%). Quanto ao ano escolar, a maior parte cursava o 7º ano do Ensino Fundamental (28,7%), seguido pelo 6º ano (26,1%) e pelo 8º ano (25,5%), sendo o 9º ano o que apresentou menor representatividade (19,7%). A maior parte dos adolescentes estava matriculada em escola de financiamento privado (79,0%).

No que diz respeito à classificação econômica, a maioria dos adolescentes pertencia à classe A1 do Critério de Classificação Econômica Brasil - CCEB (Associação Brasileira de Pesquisa, 2022) (64,5%), seguida pelas classes A2 (15,8%), B2 (8,5%), B1 (7,8%) e C1 (2,1%), sendo as classes C2 (0,7%) e D (0,7%) as menos representadas.

A análise descritiva das escalas do SDQ mostrou que a maioria dos participantes tiveram respostas nos questionários que designassem classificação normal em todas as escalas: “Sintomas Emocionais” (80,8%), “Problemas de Conduta” (79,6%), “Hiperatividade” (72,0%), “Problemas de Relacionamento com os Colegas” (80,3%) e “Comportamento Pró-Social” (89,8%). No escore total do SDQ, que indica as dificuldades em saúde mental dos participantes, 72,0% foram classificados como normais, indicando boa saúde mental. Ainda, 14,6% dos participantes apresentaram resultado limítrofe e 13,4% obtiveram pontuação classificada como anormal.

A análise descritiva dos códigos da CIF (desempenho) demonstrou que, em todos eles, a maioria dos participantes foi classificada como “sem dificuldade”, ou seja, os participantes classificaram-se capazes de desempenhar os domínios de forma efetiva, independente e funcional, sendo: d240 – sintomas emocionais (80,9%), d720 – interações interpessoais complexas (79,6%), d240 – hiperatividade (71,3%), d750 – relações sociais informais (80,3%) e d710 – interações interpessoais básicas (89,8%).

Para melhor análise de associação, a classificação do SDQ foi agrupada, a saber: a) anormal e b) limítrofe/normal. Já os grupos do CCEB, foram agrupados da seguinte forma: a) A1/A2 = A; b) B1/B2= B; e c) C1/C2/D= C-D.

A Tabela 1 apresenta a análise de associação entre as escalas do SDQ Sintomas Emocionais e Problemas de Conduta com dados sociodemográficos e códigos da CIF (desempenho), por meio dos testes Exato de Fisher e Qui-quadrado de Pearson. Observa-se que houve associação com significância estatística entre “Sintomas Emocionais” com os códigos da CIF d240 sintomas emocionais (p=0,001), d720 interações interpessoais complexas (p=0,001), d240 hiperatividade (p=0,001) e d750 relações sociais informais (p=0,001). Já para a escala Problemas de Conduta, foi observada associação com significância estatística com os códigos d240 sintomas emocionais (p=0,001), d720 (p=0,001), d750 relações sociais informais (p=0,011) e d710 interações interpessoais básicas (p=0,001). As demais análises não apresentaram resultado com significância estatística.

**Tabela 1:** Análise de associação entre as escalas sintomas emocionais e problemas de conduta do SDQ e dados sociodemográficos e CIF

**Variáveis**	**Sintomas Emocionais**			**Problemas de Conduta**		
	**Anormal N (%)**	**Limítrofe/Normal N (%)**	**valor-p**	**Anormal N (%)**	**Limítrofe/Normal N (%)**	**valor-p**
**Sexo**						
Feminino	11 (73,3)	76 (53,5)	0,142^1^	10 (62,5)	77 (54,6)	0,547^2^
Masculino	4 (26,7)	66 (46,5)		6 (37,5)	64 (45,4)	
Total	15 (100,0)	142 (100,0)		16 (100,0)	141 (100,0)	
**Ano escolar**						
6º	2 (13,3)	39 (27,5)	0,233^2^	3 (18,7)	38 (27,0)	0,550^2^
7º	7 (46,7)	38 (26,8)		7 (43,8)	38 (27,0)	
8º	2 (13,3)	38 (26,8)		3 (18,8)	37 (26,2)	
9º	4 (26,7)	27 (19,0)		3 (18,7)	28 (19,9)	
Total	15 (100,0)	142 (100,0)		16 (100,0)	141 (100,0)	
**Tipo de escola**						
Pública	2 (13,3)	31 (21,8)	0,442^1^	6 (37,5)	27 (19,1)	0,088^1^
Privada	13 (86,7)	111 (78,2)		10 (62,5)	114 (80,9)	
Total	15 (100,0)	142 (100,0)		16 (100,0)	141 (100,0)	
**CCEB**						
A	13 (86,7)	100 (79,4)	0,679^2^	10 (66,7)	103 (81,7)	0,138^2^
B	2 (13,3)	21 (16,7)		5 (33,3)	18 (14,3)	
C-D	0 (0,0)	5 (4,0)		0 (0,0)	5 (4,0)	
Total	15 (100,0)	126 (100,0)		16 (100,0)	126 (100,0)	
**d240 - SE**						
Sem dificuldade	0 (0,0)	127 (89,4)	0,001^1 *^	8 (50,0)	119 (84,4)	0,001^2^*
Com dificuldade	15 (100,0)	15 (10,6)		8 (50,0)	22 (15,6)	
Total	15 (100,0)	142 (100,0)		16 (100,0)	141 (100,0)	
**d720**						
Sem dificuldade	7 (46,7)	118 (83,1)	0,003*	0 (0,0)	125 (88,7)	0,001^1^*
Com dificuldade	8 (53,3)	24 (16,9)		16 (100,0)	16 (11,3)	
Total	15 (100,0)	142 (100,0)		16 (100,0)	141 (100,0)	
**d240 - Hiper**						
Sem dificuldade	9 (60,0)	103 (72,5)	0,307^2^	7 (43,8)	105 (74,5)	0,017^2^*
Com dificuldade	6 (40,0)	39 (27,5)		9 (56,2)	36 (25,5)	
Total	15 (100,0)	142 (100,0)		16 (100,0)	141 (100,0)	
**d750**						
Sem dificuldade	5 (33,3)	108 (76,1)	0,001^2^*	9 (56,3)	117 (83,0)	0,011^2^*
Com dificuldade	10 (66,7)	34 (23,9)		7 (43,7)	24 (17,0)	
Total	15 (100,0)	142 (100,0)		16 (100,0)	141 (100,0)	
**d710**						
Sem dificuldade	11 (73,3)	130 (91,5)	0,051^1^	10 (62,5)	131 (92,9)	0,001^2^*
Com dificuldade	4 (26,7)	12 (8,5)		6 (37,5)	10 (7,1)	
Total	15 (100,0)	142 (100,0)		16 (100,0)	141 (100,0)	

1 Teste Exato de Fisher;

2 Teste Qui-quadrado de Pearson

* valor de p≤0,05

**Legenda:** N = número de indivíduos, varia devido a dados faltantes; CCEB = Classificação Econômica Brasil; SE = Sintomas Emocionais; Hiper = Hiperatividade; d240 = Lidar com o estresse e outras exigências psicológicas; d720 = Interações interpessoais complexas; d750 = Relacionamentos sociais informais; d710 = Interações interpessoais básicas

A Tabela 2 apresenta a associação entre as escalas do SDQ Hiperatividade e Problemas de Relacionamento com os Colegas, os dados sociodemográficos e os códigos da CIF, analisados pelos testes Qui-quadrado de Pearson e Exato de Fisher. Os resultados indicaram associação estatisticamente significativa entre hiperatividade e sexo (p=0,024), com maior porcentagem de classificação limítrofe/normal entre as estudantes do sexo feminino. Também foram observadas associações significativas entre hiperatividade e os códigos da CIF: d240 – sintomas emocionais (p=0,001), d720 – interações interpessoais complexas (p=0,001), d240 – hiperatividade (p=0,001) e d710 – interações interpessoais básicas (p=0,013). Já na escala Problemas de Relacionamento, verificaram-se associações significativas com os códigos d240 – sintomas emocionais (p=0,048) e d750 – relacionamentos interpessoais informais (p=0,001). As demais associações não apresentaram significância estatística.

**Tabela 2:** Análise de associação entre as escalas sintomas de hiperatividade e problemas de relacionamento do SDQ e dados sociodemográficos e códigos da CIF

**Variáveis**	**Sintomas Hiperatividade**			**Problemas de Relacionamento**		
	**Anormal N (%)**	**Limítrofe/Normal N (%)**	**valor-p**	**Anormal N (%)**	**Limítrofe/Normal N (%)**	**valor-p**
**Sexo**						
Feminino	19 (76,0)	68 (51,5)	0,024^1 *^	3 (60,0)	84 (55,3)	0,834^2^
Masculino	6 (24,0)	64 (48,5)		2 (40,0)	68 (44,7)	
Total	25 (100,0)	132 (100,0)		5 (100,0)	152 (100,0)	
**Ano escolar**						
6º	6 (24,0)	35 (26,5)	0,988^1^	0 (0,0)	41 (27,00)	0,330^1^
7º	7 (28,0)	38 (28,8)		3 (60,0)	42 (27,6)	
8º	7 (28,0)	33 (25,0)		0 (0,0)	40 (26,3)	
9º	5 (20,0)	26 (19,7)		2 (40,0)	29 (19,1)	
Total	25 (100,0)	132 (100,0)		5 (100,0)	152 (100,0)	
**Tipo de escola**						
Pública	8 (32,0)	25 (18,9)	0,142^1^	2 (40,0)	31 (20,4)	0,290^2^
Privada	17 (68,0)	108 (81,1)		3 (60,0)	121 (79,6)	
Total	25 (100,0)	132 (100,0)		5 (100,0)	152 (100,0)	
**CCEB**						
A	15 (68,2)	98 (82,4)	0,073^1^	2 (50,0)	111 (81,0)	0,051^1^
B	7 (31,8)	16 (13,4)		1 (25,0)	22 (16,1)	
C-D	0 (0,0)	5 (4,2)		1 (25,0)	4 (2,9)	
Total	25 (100,0)	119 (100,0)		4 (100,0)	137 (100,0)	
**d240 - SE**						
Sem dificuldade	13 (52,0)	114 (86,4)	0,001^1^*	2 (40,0)	125 (82,2)	0,048^2^*
Com dificuldade	12 (48,0)	18 (13,6)		3 (60,0)	27 (17,8)	
Total	25 (100,0)	132 (100,0)		5 (100,0)	152 (100,0)	
**d720**						
Sem dificuldade	13 (52,0)	112 (84,8)	0,001^1^*	3 (60,0)	122 (80,3)	0,269^2^
Com dificuldade^1^	12 (48,0)	20 (15,2)		2 (40,0)	30 (19,7)	
Total	25 (100,0)	132 (100,0)		5 (100,0)	152 (100,0)	
**d240 - Hiper**						
Sem dificuldade	0 (0,0)	112 (84,8)	0,001^2^*	3 (60,0)	109 (71,7)	0,625^2^
Com dificuldade	25 (100,0)	20 (15,2)		2 (40,0)	43 (28,3)	
Total	25 (100,0)	132 (100,0)		5 (100,0)	152 (100,0)	
**d750**						
Sem dificuldade	17 (68,0)	109 (82,6)	0,093^1^	0 (0,0)	126 (82,9)	0,001^2^*
Com dificuldade	8 (32,0)	23 (17,4)		5 (100,0)	26 (17,1)	
Total	25 (100,0)	132 (100,0)		5 (100,0)	152 (100,0)	
**d710**						
Sem dificuldade	19 (76,0)	122 (92,4)	0,013*	3 (60,0)	138 (90,8)	0,081^2^
Com dificuldade	6 (24,0)	10 (7,6)		2 (40,0)	14 (9,2)	
Total	25 (100,0)	132 (100,0)		5 (100,0)	152 (100,0)	

1 Teste Qui-quadrado de Pearson;

2 Teste Exato de Fisher

* valor de p≤0,05

**Legenda:** N = número de indivíduos, varia devido a dados faltantes; CCEB = Classificação Econômica Brasil; SE = Sintomas Emocionais; Hiper = Hiperatividade; d240 = Lidar com o estresse e outras exigências psicológicas; d720 = Interações interpessoais complexas; d750 = Relacionamentos sociais informais; d710 = Interações interpessoais básicas

Por fim, na Tabela 3, foi realizada a análise de associação entre as escalas Comportamento pró-social e SDQ total, por meio dos testes Exato de Fisher e Qui-quadrado de Pearson, com dados sociodemográficos e códigos da CIF. Verificou-se associação com significância estatística entre Comportamento Pró-social e sexo (p=0,023), com maior porcentagem de resultado limítrofe/normal dentre os participantes do sexo feminino; e com os códigos da CIF d240 – hiperatividade (p=0,007), d750 - relações sociais informais (p=0,049) e d710 - interações interpessoais básicas (p=0,001).

**Tabela 3:** Análise de associação entre as escalas Comportamento Pró-Social e Total do SDQ e dados sociodemográficos e códigos da CIF

**Variáveis**	**Comportamento Pró-Social**			**Total SDQ**		
	**Anormal N (%)**	**Limítrofe/Normal N (%)**	**valor-p**	**Anormal N (%)**	**Limítrofe/Normal N (%)**	**valor-p**
**Sexo**						
Feminino	1 (12,5)	86 (57,7)	0,023^1 *^	15 (71,4)	72 (52,9)	0,157^1^
Masculino	7 (87,5)	63 (42,3)		6 (28,6)	64 (47,1)	
Total	8 (100,0)	149 (100,0)		21 (100,0)	136 (100,0)	
**Ano escolar**						
6º	2 (25,0)	39 (26,2)	0,236^2^	3 (14,3)	38 (27,9)	0,334^2^
7º	0 (0,0)	45 (30,2)		8 (38,1)	37 (27,2)	
8º	3 (37,5)	37 (24,8)		4 (19,0)	36 (26,5)	
9º	3 (37,5)	28 (18,8)		6 (28,6)	25 (18,4)	
Total	8 (100,0)	149 (100,0)		21 (100,0)	136 (100,)	
**Tipo de escola**						
Pública	3 (37,5)	30 (20,1)	0,366^1^	5 (23,8)	28 (20,6)	0,736^2^
Privada	5 (62,5)	119 (79,9)		16 (76,2)	108 (79,4)	
Total	8 (100,0)	149 (100,0)		21 (100,0)	136 (100,0)	
**CCEB**						
A	4 (50,0)	109 (82,0)	0,074^2^	14 (77,8)	99 (80,5)	0,552^2^
B	3 (37,5)	20 (15,0)		4 (22,2)	19 (15,4)	
C-D	1 (12,5)	4 (3,0)		0 (0,0)	5 (4,1)	
Total	8 (100,0)	133 (100,0)		21 (100,0)	123 (100,0)	
**d240 - SE**						
Sem dificuldade	5 (62,5)	122 (81,9)	0,179^1^	5 (23,8)	122 (89,7)	0,001^2^*
Com dificuldade	3 (37,5)	27 (18,1)		16 (76,2)	14 (10,3)	
Total	8 (100,0)	149 (100,0)		21 (100,0)	136 (100,0)	
**d720**						
Sem dificuldade	5 (62,5)	120 (80,5)	0,207^1^	7 (33,3)	118 (86,8)	0,001^2^*
Com dificuldade	3 (37,5)	29 (19,5)		14 (66,7)	18 (13,2)	
Total	8 (100,0)	149 (100,0)		21 (100,0)	136 (100,0)	
**d240 - Hiper**						
Sem dificuldade	2 (25,0)	110 (73,8)	0,007^1^*	7 (33,3)	105 (77,2)	0,001^2^*
Com dificuldade	6 (75,0)	39 (26,2)		14 (66,7)	31 (22,8)	
Total	8 (100,0)	149 (100,0)		21 (100,0)	136 (100,0)	
**d750**						
Sem dificuldade	4 (50,0)	122 (81,9)	0,049^1^*	7 (33,3)	119 (87,5)	0,001^2^*
Com dificuldade	4 (50,0)	27 (18,1)		14 (66,7)	17 (12,5)	
Total	8 (100,0)	149 (100,0)		21 (100,0)	136 (100,0)	
**d710**						
Sem dificuldade	0 (0,0)	141 (94,6)	0,001^1^*	13 (61,9)	128 (94,1)	0,001^2^*
Com dificuldade	8 (100,0)	8 (5,4)		8 (38,1)	8 (5,9)	
Total	8 (100,0)	149 (100,0)		21 (100,0)	136 (100,0)	

1 Teste Exato de Fisher;

2 Teste Qui-quadrado de Pearson

* valor de p≤0,05

**Legenda:** N = número de indivíduos, varia devido a dados faltantes; CCEB = Classificação Econômica Brasil; SE = Sintomas Emocionais; Hiper = Hiperatividade; d240 = Lidar com o estresse e outras exigências psicológicas; d720 = Interações interpessoais complexas; d750 = Relacionamentos sociais informais; d710 = Interações interpessoais básicas

No escore total do SDQ foi observada associação com significância estatística com com os códigos da CIF d240 – sintomas emocionais (p=0,001), d720 - interações interpessoais complexas (p=0,001), d240 – hiperatividade (p=0,001), d720 (p=0,001), d240 – hiperatividade (p=0,001), d750 - relações sociais informais (p=0,001) e d710 - relações interpessoais básicas (p=0,001).

## DISCUSSÃO

De acordo com a literatura, a aplicação clínica do SDQ foi realizada em vários países; porém, os dados normativos de significância limitam-se a poucas populações e faixas etárias^(15)^. Uma avaliação fidedigna e válida dos aspectos comportamentais dos jovens é crucial para a detecção precoce e a identificação de casos clínicos na fase infanto juvenil^(16)^. Todavia, deve-se considerar que o questionário compreende somente cinco escalas, não abrangendo todos os aspectos psicossociais de alta complexidade, especialmente na adolescência, quando ainda não se encontram completamente conscientes quanto aos seus atos^(17,18)^.

Ao observar a escala de Sintomas Emocionais do SDQ foi possível perceber associação com as escalas d240 - sintomas emocionais e d240 - hiperatividade, d720 e d750. De acordo com a literatura, adolescentes com indicadores elevados de sofrimento emocional apresentam, concomitantemente, dificuldades significativas na construção e manutenção de relações sociais, bem como na autorregulação emocional e comportamental^(19,20)^.

A associação entre a escala Problemas de Conduta e os códigos d240 - sintomas emocionais, d710, d720 e d750 demonstra que estudantes com maiores escores em problemas de conduta apresentam correlação com dificuldades expressivas em interações interpessoais, tanto no âmbito básico quanto em relações sociais mais complexas. Os comportamentos externalizantes, como agressividade, desobediência e impulsividade estão diretamente associados a déficits nas competências socioemocionais, prejudicando relações informais e a capacidade de construir e manter interações interpessoais saudáveis^(21)^. Essas dificuldades não se limitam ao convívio social, mas também impactam significativamente a capacidade do adolescente em regular suas próprias emoções e interagir de forma funcional em contextos interpessoais básicos^(22)^.

Observou-se que mais meninas do que meninos apresentam sintomas de hiperatividade. De acordo com a literatura, crianças e adolescentes do sexo feminino expressam sintomas de hiperatividade e impulsividade por meio de falas e ações, diferentemente dos meninos, que são mais rebeldes e opositores^(23)^. Como as meninas apresentam maior prevalência de traços ansiosos e depressivos, podem apresentar maiores flutuações emocionais, principalmente na faixa etária estudada^(23)^. Além disso, estudos recentes indicam que a prevalência do Transtorno de Déficit de Atenção e Hiperatividade (TDAH) é semelhante entre homens e mulheres^(24)^. Também, observou-se correlação desta escala com as categorias d240 - sintomas emocionais e d240 - hiperatividade, d710 e d720. Estudos recentes apontam que adolescentes com elevados escores na escala de hiperatividade apresentam maiores dificuldades no manejo de demandas emocionais e cognitivas^(19,20)^. A impulsividade e a inquietação dificultam habilidades sociais mais sofisticadas, como resolução de conflitos, empatia, negociação e manutenção de vínculos afetivos duradouros^(21)^. Além disso, os adolescentes podem apresentar dificuldades até mesmo nas interações cotidianas mais simples, como iniciar ou manter uma conversa e respeitar turnos de fala, comprometendo significativamente sua inserção social e o desempenho no ambiente escolar e familiar^(22)^.

Na escala Problemas de Relacionamento com os Colegas houve associação com significância estatística com as escalas d240 - sintomas emocionais e d750. Estudos atuais demonstram que adolescentes com escores elevados em problemas de relacionamento tendem a apresentar dificuldades emocionais associadas, como ansiedade social, insegurança e baixa tolerância a frustrações, aspectos diretamente relacionados à incapacidade de manejar o estresse e as demandas emocionais cotidianas^(20)^. Além disso, apresentam comprometimentos em habilidades essenciais para a convivência social, expressar-se de forma adequada ou compreender expressões faciais e sinais sociais básicos, revelando um padrão de funcionamento social disfuncional^(21)^.

O estudo em questão revelou piores resultados para o sexo masculino na escala Comportamento Pró-Social. Além disso, estes aspectos também sofrem influências socioculturais e relacionadas às práticas de socialização. No contexto brasileiro, as práticas parentais tendem a reforçar mais frequentemente comportamentos de cuidado, empatia e colaboração nas meninas. Por outro lado, os meninos são mais frequentemente direcionados a suprimir emoções e adotar posturas de resistência^(25)^. O processo de socialização desde a infância, orientado por normas culturais e estereótipos de gênero, molda como meninas e meninos lidam com emoções e relações, influenciando diretamente o desenvolvimento das competências socioemocionais e os comportamentos pró-sociais^(26)^. Também houve significância estatística na escala de comportamento e os códigos d40 - hiperatividade, d750 - relações sociais informais e d710 - interações interpessoais básicas. Adolescentes com baixos escores em comportamento pró-social apresentam dificuldades nas interações, além de enfrentarem desafios na gestão emocional, como ansiedade, frustração e impulsividade. Isso compromete sua inclusão social e bem-estar, evidenciando que o equilíbrio emocional e a competência funcional nas relações é fundamental para o desenvolvimento socioemocional^(20-22)^.

A análise do escore total do questionário SDQ evidenciou uma correlação significativa com todos os códigos da CIF analisados. A literatura destaca que adolescentes com escores elevados no SDQ tendem a apresentar maiores dificuldades em lidar com o estresse e demandas emocionais, o que impacta negativamente suas interações sociais^(25)^. Essa condição gera prejuízos no manejo emocional, na autorregulação e nas interações sociais, se manifestando como baixa tolerância à frustração, estresse constante e dificuldade de controle dos impulsos, assim como dificuldade no desenvolvimento de habilidades sociais interativas, empatia, resolução de conflitos e manutenção de vínculos^(19,20)^.

De acordo com a literatura, a CIF apresenta aplicabilidade efetiva em estudos com amostras de crianças e adolescentes. Isso se deve ao fato de a Classificação Internacional de Funcionalidade, Incapacidade e Saúde garantir um modelo padronizado e biopsicossocial que abrange as esferas social, biológica e psicológica. Esse modelo permite a identificação mais ampla de incapacidades que podem surgir ao longo do desenvolvimento^(27,28)^.

A literatura evidencia que, em sua pluralidade, adolescentes com alterações de linguagem apresentam escores do SDQ classificados como anormais^(29)^. Desse modo, a associação das escalas do SDQ com as categorias da CIF demonstrou que os adolescentes que obtiveram classificação normal no questionário não apresentaram dificuldades no desempenho. Nesse sentido, um estudo longitudinal canadense, realizado com adolescentes e jovens com condições crônicas, buscou explorar a Classificação Internacional de Funcionalidade, Incapacidade e Saúde (CIF), seus componentes funcionais e fatores contextuais, fornecendo evidências da relação entre aspectos emocionais e funcionais com a qualidade de vida^(30)^.

A Organização Pan-Americana da Saúde (OPAS) destaca que intervenções escolares são estratégias preventivas essenciais para combater condições adversas de saúde mental. Entre as ações recomendadas estão a implementação de mudanças organizacionais que promovam um ambiente psicológico seguro e positivo, a inclusão de ensinamentos sobre saúde mental e desenvolvimento de habilidades para a vida, o treinamento de profissionais para identificar e manejar riscos básicos de suicídio e a criação de programas de prevenção voltados especificamente para adolescentes vulneráveis a problemas de saúde mental^(31)^.

Este estudo apresenta algumas limitações, sendo a primeira o delineamento e a configuração amostral, com a ausência de distribuição equânime de adolescentes de escolas públicas e privadas. Desse modo, o predomínio de participantes oriundos de escolas de financiamento privado sobre o público pode ter influenciado os resultados da presente análise. Em consequência, a distribuição de participantes de acordo com a classe econômica também pode ter sido afetada, de modo que a amostragem de conveniência impede a generalização dos achados para outros contextos.

Como avanços, o estudo permitiu a possibilidade de integração entre o SDQ e a CIF, garantindo multidimensionalidade na avaliação e proporcionando um olhar mais abrangente em relação aos aspectos comportamentais e à funcionalidade no ambiente escolar e social. Além disso, a identificação das categorias associadas à CIF (comportamento pró-social, hiperatividade, problemas emocionais, de conduta e de relacionamento) facilita a formulação de intervenções mais direcionadas. O presente estudo também viabiliza a utilização da CIF no contexto escolar e cria um ponto de partida para novas pesquisas que envolvam essa faixa etária, permitindo explorar diferentes contextos, populações e intervenções baseadas nessa abordagem.

## CONCLUSÃO

Diante dos resultados, a integração entre o SDQ e a CIF se mostra uma estratégia eficaz para compreender, de forma ampla, as relações entre os aspectos emocionais, comportamentais e funcionais de adolescentes no contexto escolar. As associações encontradas reforçam que dificuldades emocionais e comportamentais impactam diretamente a funcionalidade social, o manejo do estresse e a qualidade das interações interpessoais.

Esses achados demonstram similaridades com pesquisas conduzidas no Brasil e em outros países, especialmente no que diz respeito à utilização da Classificação Internacional de Funcionalidade, Incapacidade e Saúde (CIF) para compreender aspectos comportamentais e funcionais em adolescentes. Dessa forma, fica evidente que compreender tais aspectos e suas particularidades é essencial para garantir melhor qualidade de vida emocional e social para essa faixa etária.
